# Reactivity Control of Oxidative CL-20@PVDF Composite Microspheres by Using Carbon Nanomaterials as Catalysts

**DOI:** 10.3390/ma17153805

**Published:** 2024-08-01

**Authors:** Shuwen Chen, Minghui Yu, Zhi-Hua Xue, Yibing Ding, Chao Zhang, Qi-Long Yan

**Affiliations:** 1 National Key Laboratory of Solid Rocket Propulsion, Northwestern Polytechnical University, Xi’an 710072, China; shuwenchen@nwpu.edu.cn (S.C.);; 2 School of Civil Aviation, Northwestern Polytechnical University, Xi’an 710072, China

**Keywords:** dual-oxidant, nanocarbon materials, thermal decomposition, kinetic mechanism, combustion property

## Abstract

2,4,6,8,10,12-hexanitro-2,4,6,8,10,12-hexaazaisowurtzitane (CL-20) is one of the high-energy oxidants, but has limited application due to its high sensitivity. In this work, polyvinylidene fluoride (PVDF) was used as a co-oxidizer, which is expected to increase the safety of CL-20. One kind of novel graphene-based carbohydrazide complex (GCCo and GCNi) was employed to modify the properties of dual-oxidant CL-20@PVDF composites by the spray drying method and compared with traditional nanocarbon materials (CNTs and GO). The properties of these composites were investigated using the TGA/DSC technique and impact test. The results show that GCCo and GCNi could increase the activation energy (*E_a_*) of CL-20@PVDF composites, and change the physical model of CL-20@PVDF, which followed the random chain scission model and then the first-order reaction model. In addition, these nanocarbon materials could reduce the impact sensitivity of CL-20@PVDF by their unique structure. Besides that, a dual-oxidant CL-20@PVDF system was used to improve the combustion property of Boron. GCCo and GCNi with the synergetic effect could increase the flame temperature and control the burn rate of CL-20@PVDF@B compared with CNTs and GO. The energetic nanocarbon catalyst-modified oxidant provides a facile method for stabilizing high-energy but sensitive materials to broaden their application.

## 1. Introduction

Oxidants are an important component of solid propellants. In the system of hydroxyl-terminated polybutadiene (HTPB) propellants, their oxidant content reaches over 80%. Thus, the characteristics of oxidants have a significant influence on the overall performance of solid propellants. CL-20 is one oxidant with the highest energy and density [1]. However, its insensitivity is limited to its broad applications in various fields [2]. Therefore, many researchers are focused on improving the properties of CL-20, such as crystal refinement [3], surface coating [4,5], cocrystal [6], and so on. Coating insensitive materials on the surface of CL-20 is an effective approach to improve its safety. Some researchers used polydopamine (PDA) as the shell material, which would improve the interaction energy between CL-20 and the binder [7]. Since PDA is not an energy storage material, some energetic function groups are coordinated with PDA as a modifier. PDA is always combined with kinds of metal-based energetic coordination complexes to modify the performance of CL-20 with certain properties, like reducing the laser sensitivity, increasing the safety, and so on [8,9]. PDA-nickel complex-coated multi-walled carbon nanotube complexes catalyze the decomposition of ε-CL-20 by decreasing the exothermic peak [10]. The bionic functional layer and GO could synergistically reduce the sensitivity of CL-20@PDA@GO to external stimuli [11]. Many studies show that these energetic catalyzers have great potential to modify the safety properties of energetic materials [12].

Fluorine is the most electronegative and oxidizing element [13]. Many researchers use fluorinated polymers combined with metal to improve the combustion property of metastable intermixed composite [14,15], since fluorine as an oxidizer with metal produces more gaseous products, such as metal fluorides and oxyfluorides, which are easily volatilizing. Polyvinylidene difluoride (PVDF) was easily processable to achieve homogeneous mixing with a high content of oxidizing components. PVDF could be combined with an energetic oxidant as a co-oxidant to modify the combustion behavior of metal fuel. Zuo used PVDF/CL-20 as oxidizers to accelerate the reaction process and shorten the burn time of silicone powder [16]. PVDF was also composed of AP to improve the combustion efficiency of Al [17]. Thus, it is promising that PVDF is used as a co-oxidizer with common oxidizers in solid propellant, resulting in a higher heat release. Jing calculated the binding energy of CL-20 and fluorine adhesives with COMPASS force filed by Materials Studio, and found that CL-20 has a large binding energy with fluorine adhesives, which would have better compatibility [18]. Huang also used molecular dynamics to study the interface systems of 3-nitro-1,2,4-triazole-5-one (NTO) and fluoropolymer binders, it detected that PVDF reduced the acidity of NTO, and significantly improved its compatibility and safety [19]. Since adding oxidants into the solid propellant is an effective way to enhance the afterburning reactions and increase the total energy, it is very important and meaningful to study the influence of catalysts on oxidants.

A large amount of research has been conducted on the application of carbon materials and their composites in oxidants, especially carbon nanotubes (CNTs), graphene (GO, rGO), and their composites as catalysts. They have a large specific surface area, which endows them with good thermal conductivity. CNTs are nanomaterials composed of a two-dimensional hexagonal lattice of carbon atoms, which bends in one direction and combines to form a hollow cylinder. By utilizing the porous structure and easy adsorption characteristics of carbon nanotubes, physical or chemical methods are used as carriers for combustion catalysts to load nanocatalysts onto carbon materials to form a composite. The single-walled carbon nanotubes (SWCNTs) and the multi-walled carbon nanotubes (MWCNTs) were discussed on the catalytic activity of ammonium perchlorate (AP), both showed a catalyst effect on the decomposition of AP with a lower activation energy and higher heat release [20]. The presence of MWCNTs coated by CuO reduced the activation energy of 1,3,5-trinitro-1,3,5-triazinane (RDX) [21]. Cyclotetramethylenetetranitramine (HMX) particles were homogeneously distributed on the surfaces of the CNTs, resulting in better thermal conductivity and mechanical sensitivity than pure HMX [22]. The laser ignition performance of NC-NG-RDX propellants (nitrocellulose, NC; nitroglycerine, NG) doped with CNTs improved obviously because of the high laser absorption property of CNTs, which revealed that CNTs can be used as a laser sensitizer [23].

Compared with CNTs, GO has a single-layer structure with several oxygen-containing functional groups, which has a large specific surface area. Moreover, a series of GO-based composites are obtained by combining them with other metals, metal oxides, and other substances. This composite sometimes produces synergistic effects to obtain more excellent properties. To improve the safety of HMX, GO could decrease its impact and friction sensitivity, and improve its thermal stability as well [24]. Wang studied the mixture of rGO and CNTs, which formed a three-dimensional heat-conducting network structure, which result in better thermal conductivity on CL-20-based composites than rGO or CNTs alone [25]. The synergistic effect between GO and Bi_2_WO_6_ has a catalytic activity on the thermal decomposition of RDX [26]. GO-doped transition metal complexes of triaminoguanidine (TAG) have the catalytic reactivity on thermolysis of RDX and improved its thermal stability at the same time [27]. Liang used GO/Cu-MOF (metal–organic frameworks, MOF) to modify the thermal decomposition performance of AP. The activation energy was decreased and the heat release was increased almost four times compared with pure AP [28]. Feng synthesized a new energetic coordination compound GO-Co-AMTZ (nitrogen-rich 3-amino-1,2,4 triazole, AMTZ), which has good thermal stability and catalytic effect on the AP decomposition process [29]. GO-TAG could reduce the mechanical sensitivity and increase the heat release of ε-CL-20 [30].

In this study, PVDF was used as a co-oxidant with CL-20. The direct catalytic effect of nanocarbon materials on the dual-oxidizer CL-20@PVDF should be considered. Thereby, the nanocarbon materials containing CL-20@PVDF composites have been prepared and evaluated. In addition, several nanocarbon additives were composed with them, to evaluate their influence on the thermal and mechanical properties of CL-20@PVDF. Moreover, nanocarbon additives doping CL-20@PVDF were used to evaluate the combustion behavior of Boron. This work chose four kinds of nanocarbon: graphene oxide, carbon nanotubes, and graphene-based energetic coordination polymers. The morphology, thermal properties, kinetic mechanism, impact sensitivity, and combustion performance were investigated in detail.

## 2. Experimental Section

### 2.1. Materials

Raw CL-20 was provided by the Xi’an Modern Chemistry Research Institute (Xi’an, China). The PVDF (Kynar 761, with an average M.W. of 550,000) was sold by Arkama company (Colombes, France). The commercial GO (1–3 layers, oxygen content > 42%) was produced by Nanjing Jicang Nano Technology Co. (Nanjing, China). The carboxylic multi-walled carbon nanotubes (CNTs) were purchased from XFNANO (Nanjing, China). The GO-CHZ-Co (GCCo) and GO-CHZ-Ni (GCNi) were prepared following the method in the published paper [31], and their property was tested by reference [32]. *N*,*N*-dimethylformamide (DMF, 99.5%) was obtained from Beijing Chemical Reagent Company (Beijing, China). Micron boron powder was bought from Liaobing Chemical Company (Yingkou, China).

### 2.2. Preparation of CL-20@PVDF and CL-20@PVDF@B Microspheres

**Figure 1:**
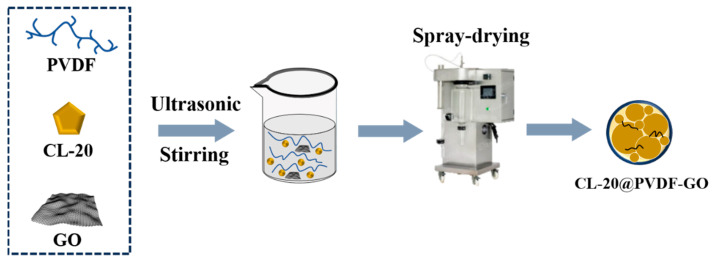
Scheme of preparation of CL-20@PVDF microspheres employed by spray drying method (GO as a catalyzer was a sample).

To attain the optimized morphology and a better understanding of the interaction mechanism between two components, the weight ratio of CL-20/PVDF was kept at 2:1. The ratio of nanocarbon was 1 wt% to investigate their influence on CL-20@PVDF microspheres. Firstly, 1 g PVDF was dissolved in 50 mL DMF with the effect of magnetic stirring at 40 °C, and 0.03 g nanocarbon was suspended in 15 mL DMF by ultrasonic at the same time. After 4 h, these two solutions were mixed, and then 2 g CL-20 was added to the above mixture solution. Finally, the solution was fed into a spray dryer (YC-015, Pilotech, Shanghai, China). The feed rate was set as 5 mL/min, and the inlet temperature was 135 °C. By using the spray drying method, CL-20@PVDF composites were collected in a connected glass collector. Figure 1 shows the preparation process of CL-20@PVDF microspheres. These microspheres modified by nanocarbon were named CL-20@PVDF, CL-20@PVDF-GO, CL-20@PVDF-CNT, CL-20@PVDF-GCCo, and CL-20@PVDF-GCNi, respectively. The CL-20/PVDF-1 was made by the weight ratio of CL-20/PVDF is 1:1 as a reference.

The CL-20@PVDF@B composite particles (CPB) were prepared by the same method as CL-20@PVDF. The weight ratio of CL-20/PVDF/B is 2:1:2.

### 2.3. Characterization Techniques

The morphology and structure of CL-20@PVDF microspheres were examined by using a scanning electron microscope (SEM, Quanta FEG 250, Houston, TX, USA). The SEM image was used to measure the particle size by ImageJ 1.8.0. Each sample was measured around five hundred times. The thermal property of samples was detected by using the TGA-DSC technique (HCT-3, HENVEN Ltd, Beijing, China). The samples were tested in an alumina pan with a pin-hole cover in a temperature range of 50–600 °C under an argon atmosphere (gas flow rate: 50 mL min^−1^). The heating rates were set to 5, 10, 15, and 20 K min^−1^, respectively. The impact sensitivity was examined by the GJB 772A-97 [33] explosive test method, and each sample of the impact sensitivity was tested 25 times with a drop hammer of 2 kg. The combustion performance of propellant samples was recorded by the high-speed camera (Phantom M340, San Francisco, CA, USA) and high-speed infrared thermal camera (FLIR X6530sc, Wilsonville, OR, USA). The microspheres were ignited with an electrically heated Ni/Cr filament (0.5 mm in diameter) by a discharge current of 5 A.

## 3. Results

### 3.1. Morphology Characterization of Samples

**Figure 2:**
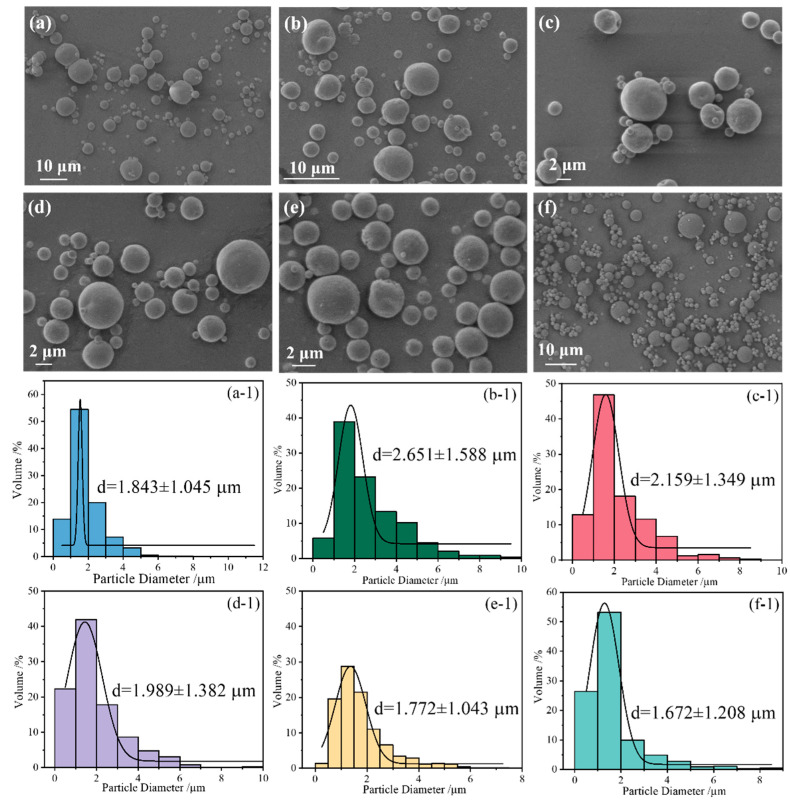
The images of CL-20@PVDF microspheres ((**a**) CL-20@PVDF; (**b**) CL-20@PVDF-CNT; (**c**) CL-20@PVDF-GO; (**d**) CL-20@PVDF-GCCo; (**e**) CL-20@PVDF-GCNi; (**f**) CL-20@PVDF-1 and the weight ratio of CL-20: PVDF = 1:1) and size distributions ((**a-1**) CL-20@PVDF; (**b-1**) CL-20@PVDF-CNT; (**c-1**) PVDF-GO; (**d-1**) CL-20@PVDF-GCCo; (**e-1**) CL-20@PVDF-GCNi; (**f-1**) CL-20@PVDF-1).

To investigate the microscopic morphology and particles’ size of nanocarbon-doped CL-20@PVDF, the morphology of involved CL-20@PVDF particles was characterized by SEM. Figure 2 shows that the composite particles are spherical structures with smooth surfaces. It is assumed that most nanocarbon is wrapped inside CL-20@PVDF and the diameters are 1.672–2.651 μm. When the weight ratio of CL-20 and PVDF is 1:1, there are several small particles existing (Figure 2f). Comparing Figure 2f with Figure 2a, the particle size decreases when the content of PVDF increases. Thus, the weight ratio of CL-20:PVDF is fixed as 2:1 for better-optimized morphology.

By using the spray drying method, PVDF would mix with CL-20 to form the spherical composite particles. However, the conductivity or boiling point of the suspension has been altered by the addition of nanocarbon materials. Therefore, the particle size distribution has been changed, and some particles with rough surfaces, although the same technology parameters were used in the preparation process (Figure 2b–e). As shown in Figure 2b,c, the particle diameters increase under the influence of CNTs and GO. In contrast, GCCo has a slight effect on increasing the particle size, while GCNi has the opposite effect. In the spray drying method, the suspension was atomized to form a large number of droplets. Then, at a higher temperature, the solvent in the droplet would volatilize and evaporate, and solid particles would be formed. The existence of nanocarbon materials in the suspension will influence the surface interactions between the component molecules. Finally, nanocarbon-modified CL-20@PVDF composites with a range of particle sizes were prepared.

### 3.2. Thermal Stability of Samples

**Figure 3:**
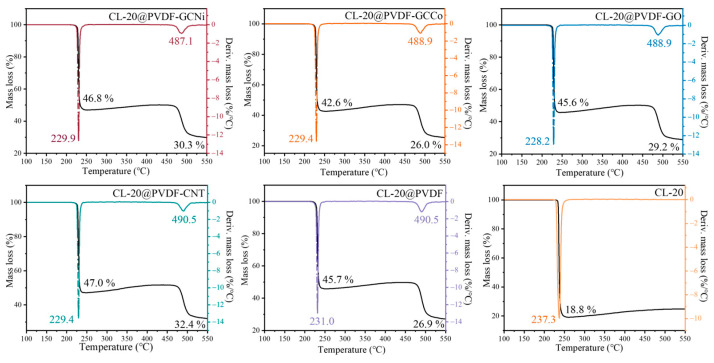
Schemes TGA/DTG of CL-20@PVDF microspheres at 10 K/min.

**Figure 4:**
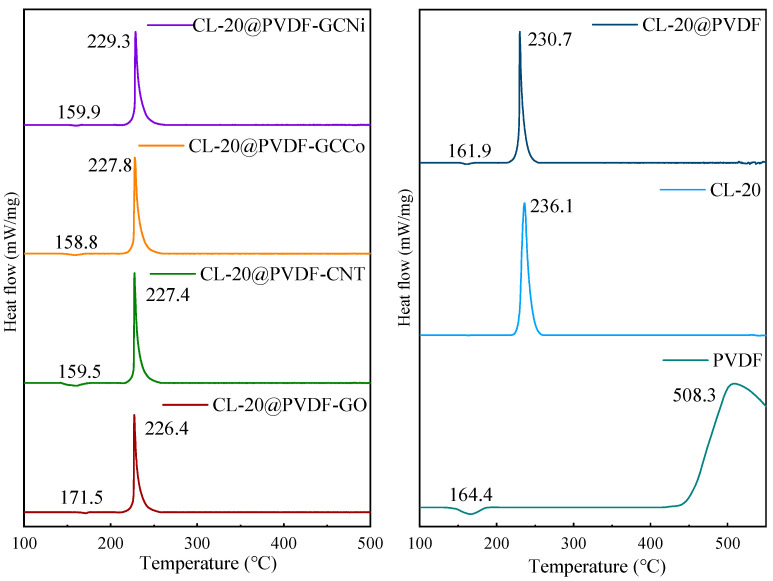
DSC curves of CL-20@PVDF microspheres at 10 K/min.

The thermal behaviors of the involved CL-20@PVDF microspheres were measured by the TGA-DSC technique. The mass loss and heat flow curves are presented in Figure 3 and Figure 4, and the corresponding parameters are summarized in Table 1 and Table 2. The thermal decomposition of PVDF is in Figure S1 and its peak temperature of the DTG curve is 477.9 °C. In Figure 3, the onset decomposition temperature of CL-20 and CL-20@PVDF microspheres were 227.6 °C and 213.3 °C, respectively. It has been suggested that PVDF forward the onset temperature of CL-20 degradation. This demonstrates the dual role of PVDF in the CL-20@PVDF system, promoting the thermal process of CL-20 and acting as an oxidizer.

In the TGA/DTG curves (Figure 3), there are two steps for the decomposition of CL-20@PVDF microspheres, which are correlated with the decomposition of CL-20 and PVDF individually. The peak temperatures of CL-20@PVDF are 231.0 and 490.5 °C, which are 6.3 and −12.6 °C earlier than those of pure CL-20 and PVDF. It can be assumed that each component affects the pyrolysis process of the other. The nanocarbon materials have an accelerated effect on the decomposition of composite particles. In the first stage of the mass loss process, GO has a more obvious catalytic effect on CL-20 thermal decomposition compared with other additives. For the second stage, GCNi decreases the peak temperature by 3.4 °C, which has the maximum influence on the decomposition of PVDF. The addition of nanocarbon materials could catalyze the two decomposition steps of CL-20@PVDF microspheres by 1.1–2.8 °C and 1.6–3.4 °C, respectively. The value of the mass loss for each step in five composites remains almost the same, suggesting that the content ratio of each component in the particles is consistent.

In the DSC curve of CL-20@PVDF (Figure 4), there is one endothermic peak at around 160 °C, which represents the melting of PVDF. An exothermic peak at 236.1 °C followed, owing to the thermal decomposition of CL-20. With the same result as DTG, the addition of PVDF can catalyze the decomposition of CL-20 by reducing the initial and peak temperatures. It assumed that the presence of PVDF may have a dissolving effect on decreasing crystal lattice stabilization [34]. Concerning the influence of nanocarbon materials, GO has a stable effect on the endothermic process, whereas the others would catalyze this process. In the case of the exothermic reaction of CL-20@PVDF, four kinds of nanocarbon materials have a catalytic influence on the decomposition of CL-20@PVDF by decreasing the peak temperature of the exothermic reaction. Additionally, all four types of nanocarbon materials could prolong the reaction process of CL-20 by increasing the temperature interval. It is suggested that the additive would slow down the heat release process.

### 3.3. Kinetics and Mechanisms of CL-20@PVDF Microspheres

To further understand the thermal mechanism of nanocarbon-modified CL-20@PVDF microspheres, their thermal decomposition kinetics were investigated using the exothermic peak in the DSC curves. The DSC curves of the relevant composites obtained at four heating rates (5, 10, 15, and 20 K min^−1^) are shown in Figure S7. Using the methods shown in the Supporting Information (Section S1), *E_a_* was calculated using the Kissinger, Friedman, and combined kinetic methods (Equations (S6)–(S8) in Supporting Information), respectively. The detailed kinetics parameters, including activation energy (*E_a_*), pre-exponential factor (*A*), correlation coefficient (*r*), and exponential factors (*m* and *n*) are summarized in Table 3. As previously mentioned, the endothermic peak in the DSC curve comprises two reactions: the melting process of PVDF and the polymorphic transition of CL-20. Therefore, we would use the exothermic peak around 230 °C to calculate and clarify the reaction mechanism of CL-20@PVDF. The kinetic parameters of this exothermic peak is used to compare the effect of nanocarbon on the decomposition of CL-20@PVDF.

In the Kissinger method, the activation energy of CL-20@PVDF is 167.3 kJ mol^−1^. After doping various kinds of nanocarbon materials into the CL-20@PVDF samples, a higher *E_a_* was observed compared with CL-20@PVDF. Especially for GO-doped transition metal complexes, *E_a_* of the exothermic peak for CL-20@PVDF-GCCo and CL-20@PVDF-GCNi is 270.6 and 267.5 kJ mol^−1^, which were increased by 103.3 and 100.2 kJ mol^−1^ in comparison to that of CL-20@PVDF. In the presence of nanocarbon materials, the energy barrier of the thermolysis reaction is increased, which leads to better stability of CL-20@PVDF. However, it notes that the Kissinger method only focuses on the peak temperature, so it is not accurate if the reaction process contains several steps.

**Figure 5:**
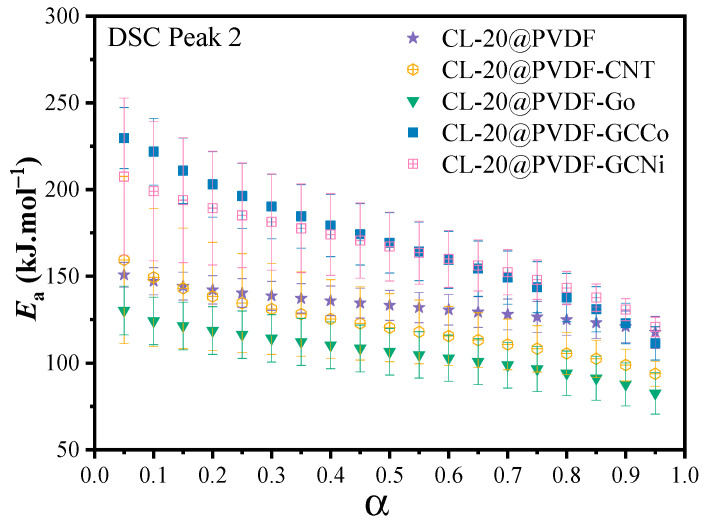
The dependence of *E_a_* on the extent of conversion (α) for the DSC curve of CL-20@PVDF microspheres.

Since the reaction process of energetic materials is always followed by an n-order reaction, other more precise calculations would be used. The Friedman method has been used to express the relationship between the conversion rate (α) and activation energy (*E_a_*). As shown in Figure 5, the *E_a_* value of involved CL-20@PVDF particles decreases as the conversion rate increases, which suggests that the autocatalytic reaction occurred during the thermal decomposition process. For the degree of conversion of α < 0.15, the apparent activation energy of CL-20@PVDF-CNTs is larger than that of CL-20@PVDF, then *E_a_* keeps a lower value with increasing α. The *E_a_* values of CL-20@PVDF-GO were reduced compared with CL-20@PVDF in the process. For CL-20@PVDF-GCCo and CL-20@PVDF-GCNi, the *E_a_* values are very high initially and then drop rapidly. In order to compare the catalytic effect of different additives on CL-20@PVDF decomposition, the average *E_a_* (0.3 < α < 0.7) was obtained, and the results are shown in Table 3. The *E_a_* values of CL-20@PVDF-CNT, CL-20@PVDF-GO, CL-20@PVDF-GCCo, and CL-20@PVDF-GCNi were 120.5, 106.6, 169.4, and 167.1 kJ mol^−1^, respectively. It increases by −12.8, −26.7, 36.1, and 33.8 kJ mol^−1^ compared with CL-20@PVDF. For the Friedman method, the presence of CNTs and GO would reduce the *E_a_*; however, GCCo and GCNi showed a reverse effect. For CL-20@PVDF-GCCo and CL-20@PVDF-GCNi, their value difference of *E_a_* was too large as the change of α. Thus, the combined kinetic method is used to evaluate the reaction model of the composites.

**Figure 6:**
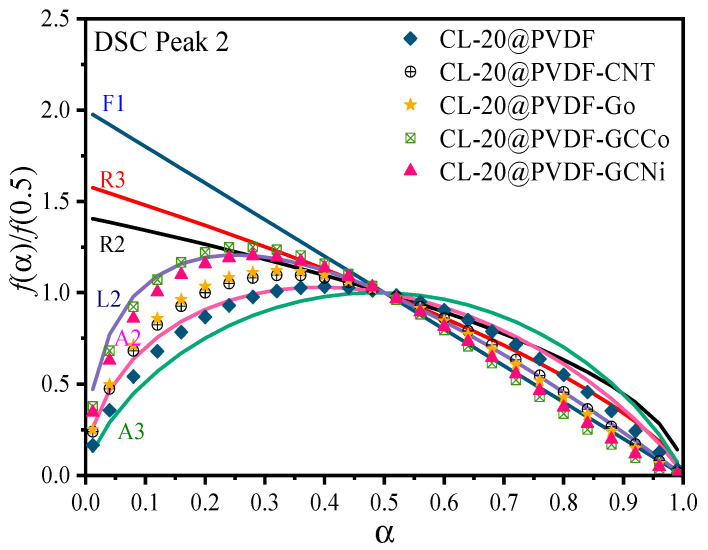
A comparison of the kinetic models for DSC curves of CL-20@PVDF microspheres obtained by a combined kinetic analysis method with the ideal models. Notes: F1, first order reaction, so-called unimolecular decay law, where random nucleation followed by an instantaneous growth of nuclei; R2, phase boundary controlled reaction (contracting area), R3, phase boundary controlled reaction (contracting volume); L2, random chain scission model; A2, A3, random two and three-dimensional nucleation and nucleus growth models.

By using the combined kinetic method, the kinetic parameters of the composite particles involved can be obtained in Table 3, and their physical models are plotted in Figure 6. Except for GO, the other three additives would increase the *E_a_* of CL-20@PVDF. The ideal model of CL-20@PVDF was the random two-dimensional nucleation and nucleus growth (A2) model, which then switched to the random chain scission (L2) model. After doping with CNTs and GO, the thermal decomposition model of CL-20@PVDF samples was still consistent with the A2 and then L2 models. It shows that the introduction of CNTs and GO did not alter their decomposition model. When α < 0.5, the scattered reaction would occur on the surface of the composite particles initially, and then the polymer chain scission reaction would determine their degradation rate.

The thermal decomposition kinetics of CL-20 is controlled by N-NO_2_ bond breaking, of which the thermal decomposition gas product is mainly NO_2_ [35]. After the addition of carbon nanotubes and graphene oxide, more surface-active sites can be formed due to the more active end groups on their surface, which react with the NO_2_ and promote the cleavage of the N-NO_2_ radicals. It demonstrated that the starting decomposition temperature and activation energy are lowered. The reaction between them releases a large amount of heat and accelerates the bond-breaking reaction of CL-20 so that the decomposition continues [36].

In the AP/PVDF system [17], the types of metal ions play an important influence on the change of kinetic models. The decomposition model of AP/PVDF would be changed depending on the types of metal ions. In contrast, CL-20/PVDF is less sensitive to the kinds of metal ions. The decomposition of CL-20@PVDF-GCCo and CL-20@PVDF-GCNi matched well with the L2 model primarily, which means that the energetic catalyst could suppress the decomposition of CL-20, and the polymer chain scission dominated the reaction [37,38]. When the thermal decomposition process at α > 0.5, small molecule clusters were formed as the long chain was broken. Therefore, the reaction rate was proportional to the reactant concentration which followed the first-order reaction model (F1). Due to the existence of a carbon skeleton, it would prevent the agglomeration of the metal. Then the metal ion becomes the reactive center, which mainly affects the pyrolysis properties [39].

### 3.4. Impact Sensitivity of CL-20@PVDF Microspheres

The impact sensitivity is one important factor to measure the safety of explosives and the results are summarized in Table 4.

As shown in Table 4, the impact sensitivity has been reduced by adding nanocarbon materials. The unique structure of GO and CNTs offers excellent thermal conductivity and mechanical properties for composite particles. These properties would lower the formation of hotspots, resulting in a reduction in the impact sensitivity of CL-20@PVDF. Compared to CL-20@PVDF, CL-20@PVDF-CNT has an impact sensitivity of 4.0 J, a 33.3% reduction, whereas CL-20@PVDF-GO exhibits an impact sensitivity of 6.0 J with a 100% decrement. It showed that the layered structure of GO would exhibit superior deformation resistance. The presence of GCCo and GCNi still reduced the impact sensitivity of the CL-20@PVDF. As both GCCo and GCNi contain the energetic functional group inside, the impact sensitivity decreases by 33.3% and 20%, respectively.

### 3.5. Burn Rate and Flame Temperature of CL-20@PVDF@B Microspheres

**Figure 7:**
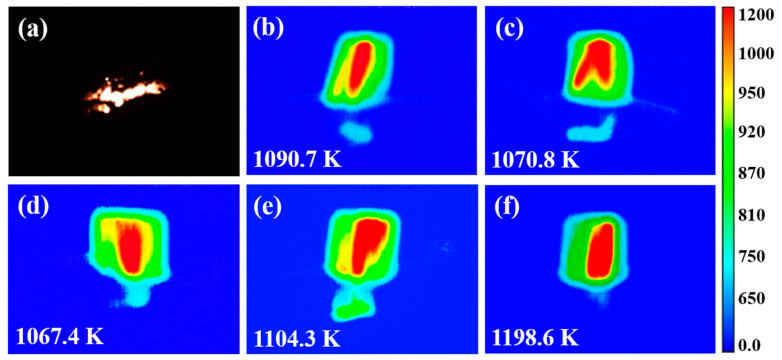
The flame images of involved CL-20@PVDF@B (**a**) and the infrared flame image in the highest temperature during the combustion process (**b**) CL-20@PVDF@B; (**c**) CL-20@PVDF@B-CNTs; (**d**) CL-20@PVDF@B-GO; (**e**) CL-20@PVDF@B-GCCo; (**f**) CL-20@PVDF@B-GCNi.

Boron has a higher volumetric calorific value, but its oxygen consumption is also extremely high. Boron powder has several problems such as difficult ignition and low combustion efficiency under engine conditions. Thus, the dual-oxidant CL-20@PVDF system was used to evaluate the combustion performance of boron powder. The nanocarbon materials modified CL-20@PVDF@B microspheres (CPB) were prepared by the spray-drying method. The flame propagation processes of the relevant composite particles were recorded by the high-speed camera and high-speed infrared thermal camera in the combustion diagnostic chamber. All the powders were placed in a cylindrical hollow quartz tube. The loading mass is around 280 mg for each test. The flame sequence images are shown in Figure 7 and Figure S8, and the burn rates are summarized in Table 5.

As shown in Figure 7a and Figure S8, the flame color of all the involved composite particles is bright yellow, and a lot of black solid combustion products were generated on the tube wall. For the blank sample CL-20@PVDF@B, the burn rate is 19.89 mg s^−1^. The additive of nanocarbon materials would increase the burn rate of CL-20@PVDF@B, since they have excellent thermal conductivity. The burn rate of CL-20@PVDF@B-CNTs increases to 24.44 mg s^−1^, with a 22.9% increment. However, the burning rates of graphene-based additives are slower than CL-20@PVDF@B-CNTs, resulting in their structure dissimilarity. The heat transfer could benefit more from the tube structure than the layer structure. For graphene-based additives, the GCCo and GCNi would reduce the burn rate of CL-20@PVDF@B compared with CL-20@PVDF@B-GO. It may be attributed to the synergetic effect between the catalytic effect from the metal ions and the stabilizing effect from the CHZ ligand, which corresponds to graphene-based carbohydrazide complexes modified Al@PVDF/AP system [17].

We chose the image where the highest temperature appeared, then we found that the addition of CNTs and GO (Figure 7c,d) could reduce the highest flame temperature to 1070.8 K and 1067.4 K from 1090.7 K of CL-20@PVDF@B, respectively. However, the graphene-based energetic catalyst would increase the highest flame temperature to 1104.3 K and 1198.6 K (Figure 7e,f). This is because the energetic compound could increase the flame temperature. Meanwhile, the CNTs and GO without energy would decrease the flame temperature. The results indicate that nanocarbon materials show a catalytic effect on the flame propagation process of CL-20@PVDF@B of their unique structure. The graphene-based carbohydrazide complexes would increase the flame temperature, and have a better potential of controlling the burning rate. Therefore, graphene-based carbohydrazide complexes could be a promising alternative with higher combustion efficiency when used in propellants.

## 4. Conclusions

In this paper, nanocarbon (CNTs, GO, GCCo, and GCNi) was used to modify the thermal and combustion properties of the dual-oxidant CL-20@PVDF and CL-20@PVDF@B microspheres by the spray drying method. The decomposition process, thermal mechanism, and impact sensitivity of different composites were investigated and compared. Meanwhile, the combustion property of dual-oxidant CL-20@PVDF improved boron was also studied. The conclusions are achieved as follows:

(1) For CL-20@PVDF dual-oxidant particles, PVDF can promote the degradation of CL-20. Four different types of nanocarbon materials all show an accelerated effect on the decomposition of CL-20@PVDF microspheres. Among all the additives, GO shows a more obvious catalytic effect on CL-20 thermal decomposition, and GCNi presents the maximum acceleration effect on the decomposition of PVDF.
(2) In the Kissinger method, the doping of nanocarbon causes the increase of activation energy for CL-20@PVDF. For the Friedman method, the *E_a_* value of involved CL-20@PVDF particles decreases as the conversion rate increases, which suggests that the autocatalytic reaction occurred. By using the combined kinetic method, the additive of CNTs and GO did not change the decomposition model of CL-20@PVDF, all their kinetic models remained A2 and then L2 model. However, CL-20@PVDF-GCCo and CL-20@PVDF-GCNi followed L2 and then the F1 model.
(3) Since nanocarbon additives have superior thermal conductivity and deformation resistance, they reduce the impact sensitivity of CL-20@PVDF particles, wherein GO could reduce the impact sensitivity by 100%.
(4) The additive of nanocarbon materials would increase the burn rate of CL-20@PVDF@B, since they have excellent thermal conductivity. Moreover, graphene-based carbohydrazide complexes could increase the flame temperature and control the burn rate with their synergetic effect. It shows that the introduction of graphene-based energetic complexes could lead to higher combustion efficiency.

## Figures and Tables

**Table 1:** The non-isothermal TG/DTG data of CL-20@PVDF microspheres (heating rate 10 K min^−1^).

Samples	TG Curves			DTG Peaks	
	*T_i_*/°C	Mass Loss/%	*L_max_* %*/*°C	*T_p_*/°C	*T_i_*/°C
CL-20	227.6	81.2	15.6	237.3	233.8
CL-20@PVDF	213.3/463.3	54.3/18.8	10.7/0.87	231.0/490.5	228.9/482.5
CL-20@PVDF-CNT	213.7/463.3	53.0/14.6	10.4/0.72	229.4/490.5	226.5/480.0
CL-20@PVDF-GO	209.2/462.1	54.4/16.4	11.1/0.67	228.2/488.9	226.1/476.9
CL-20@PVDF-GCCo	214.4/463.3	57.4/16.6	11.3/0.82	229.4/488.9	226.5/475.9
CL-20@PVDF-GCNi	213.2/459.8	53.2/16.5	11.0/0.75	229.9/487.1	227.5/474.8

Note: *T_i_*—the initial temperature for thermal decomposition; mass loss—from the initial temperature to the end temperature of DTG peak; *L_max_*—the maximum mass loss rate; *T_p_*—the peak temperature of mass loss rate.

**Table 2:** Detailed comparison of DSC data for CL-20@PVDF microspheres (10 K/min).

Samples	Exothermic Peaks (10 K min^−1^)			
	*T_o_*/°C	*T_p_*/°C	*T_e_/*°C	∆*T*
CL-20	216.4	236.1	261.6	45.2
CL-20@PVDF	153.4/210.4	161.9/230.7	169.5/254.8	16.1/44.4
CL-20@PVDF-CNT	149.2/212.1	159.5/227.4	169.3/256.6	20.1/44.5
CL-20@PVDF-GO	165.7/209.8	171.5/226.4	174.8/255.9	9.1/46.1
CL-20@PVDF-GCCo	153.0/208.9	158.8/227.8	168.7/259.4	15.7/50.5
CL-20@PVDF-GCNi	154.7/214.2	159.9/229.3	164.8/259.3	10.1/45.1

Note: *T_o_*, the onset temperature; *T_p_*, the peak temperature of thermal events; *T_e_*, the end temperature for heat change; ∆*T*, temperature interval.

**Table 3:** The kinetic parameters for the decomposition reaction of CL-20@PVDF microspheres.

Samples	Combined Kinetic Method			Friedman Method			Kissinger Method		
	*m*	*n*	*E_a_*_(1)_	*A*/min^−1^	*E_a_*_(2)_	*r*	*E_a_*_(3)_	log *A*	*r*
CL-20@PVDF	0.665	0.993	130.9 ± 1.2	4.6 × 10^14^	133.3	0.9958	167.3	11.6	0.9916
CL-20@PVDF-CNT	0.586	1.163	135.2 ± 3.5	1.3 × 10^15^	120.5	0.9766	211.2	16.2	0.9896
CL-20@PVDF-GO	0.597	1.238	116.1 ± 3.8	1.1 × 10^13^	106.6	0.9734	233.9	18.6	0.9881
CL-20@PVDF-GCCo	0.527	1.453	171.5 ± 5.1	7.5 × 10^18^	169.4	0.9671	270.6	22.5	0.9740
CL-20@PVDF-GCNi	0.532	1.350	161.1 ± 2.2	4.9 × 10^17^	167.1	0.9875	267.5	22.1	0.9777

Notes: *m* and *n* are the exponential factors of the combined kinetic method; *E_a_*_(1)_, *E_a_*_(2)_, and *E_a_*_(3)_ are the average activation energy calculated by an isoconversional method, Friedman, Kissinger, and combined kinetic method, respectively, in kJ mol^−1^; *A*, the pre-exponential factor, in min^−1^; *r*, correlation coefficient.

**Table 4:** The impact sensitivity results of involved samples.

Samples	Impact Sensitivity (Im, J)
CL-20@PVDF	3.0
CL-20@PVDF-CNT	4.0
CL-20@PVDF-GO	6.0
CL-20@PVDF-GCCo	4.0
CL-20@PVDF-GCNi	3.6

**Table 5:** Burn rates for the involve CL-20@PVDF@B (CPB) microspheres.

Samples	CPB	CPB-CNTs	CPB-GO	CPB-GCCo	CPB-GCNi
Burn time (s)	11.85	12.06	13.35	13.55	13.04
Burn rate (mg s^−1^)	19.89	24.44	21.85	20.30	21.53

## Data Availability

The original contributions presented in the study are included in the article/supplementary material, further inquiries can be directed to the corresponding author.

## Supplementary Materials

The following supporting information can be downloaded at: https://www.mdpi.com/article/10.3390/ma17153805/s1, Figure S1: TGA/DTG and DSC of PVDF at 10 K/min; Figure S2: TGA/DTG of CL-20@PVDF microspheres at 5, 10, 7.5, 15 K/min; Figure S3: TGA/DTG of CL-20@PVDF-CNT microspheres at 5, 10, 15, 20 K/min; Figure S4: TGA/DTG of CL-20@PVDF-GO microspheres at 5, 10, 15, 20 K/min; Figure S5 TGA/DTG of CL-20@PVDF-GCCo microspheres at 5, 10, 15, 20 K/min; Figure S6: TGA/DTG of CL-20@PVDF-GCNi microspheres at 5, 10, 15, 20 K/min; Figure S7: DSC of involved CL-20@PVDF microspheres at 5, 10, 15, 20 K/min; Figure S8: Flame propagation images of the combustion processes of involved CL-20@PVDF@B: (a) CL-20@PVDF@B; (b) CL-20@PVDF@B-CNTs; (c) CL-20@PVDF@B-GO; (d) CL-20@PVDF@B-GCCo; (e) CL-20@PVDF@B-GCNi. References [40,41,42,43,44,45,46] are cited in the supplementary materials.
