# Supplementary material for: Reactivity Control of Oxidative CL-20@PVDF Composite Microspheres by Using Carbon Nanomaterials as Catalysts
Source: Materials (Basel). 2024 Aug 1;17(15):3805. doi: 10.3390/ma17153805 (PMC11313176; doi:10.3390/ma17153805)
Supplement: Supplementary file 1 [file materials-17-03805-s001.zip › materials-3067072-supplementary.pdf]

Reactivity Control of Oxidative CL-20@PVDF Composite Microspheres by Using Carbon Nanomaterials as Catalysts

Shuwen Chen [1], Minghui Yu [1], Zhi-Hua Xue [1], Yibing Ding [1], Chao Zhang [2,*] and Qi-Long Yan [1,*]

1. National Key Laboratory of Solid Rocket Propulsion, Northwestern Polytechnical University, Xi'an 710072, China

2. School of Civil Aviation, Northwestern Polytechnical University, Xi'an 710072, China

1. Kinetic theory

Since the components are complicated and their decomposition processes are complex, one simple kinetic method cannot describe the composites' pyrolysis. In this study, the decomposition kinetics is investigated by the Kissinger, Friedman, and combined kinetic methods. Peakfit and Mathcad software was employed to calculate the kinetic parameters for the whole thermolysis process.

The reaction conversion (α) is always a variable with relevant conditions, and its value ranges from 0 to 1. To simplify the complex pyrolysis, the reaction conversion rate (Eq. (S1)) is expressed by the product of the reaction model function *f(α)* and the constant rate *k(T)* [1].

$$\frac{d\alpha}{dt} = f(\alpha)k(T) \qquad \text{(S1)}$$

The most used functions are the *n*-order model, Eq. (S2), and the autocatalytic one, Eq. (S3) [2].

$$f(\alpha) = (1-\alpha)^n \qquad \text{(S2)}$$

$$f(\alpha) = \alpha^m(1-\alpha)^n \qquad \text{(S3)}$$

Order n reaction is valid when the reaction products do not affect the reaction rate, while autocatalytic reaction is valid when the reaction rate is accelerated by the reaction products. In order *n* reaction, it assumes that there is one reaction that happened. For the autocatalytic model, the reaction rate is changed during the decomposition process.

The temperature dependence *k (T)* is commonly described by the Arrhenius equation [3], Eq. (S4):

$$k(T) = Aexp[-E_a/(RT)] \qquad \text{(S4)}$$

Under constant heating, the *k (T)* in Eq. (S1) may be eliminated and $\beta = dT/dt$, then Eq. (S5) can be expressed by:

$$\frac{d\alpha}{dT} = \frac{A}{\beta}\exp\left(-\frac{E}{RT}\right)f(\alpha) \qquad \text{(S5)}$$

Kissinger's method [4] assumed that the reaction rate only depends on the temperature, and the reaction order *n* was 1. The kinetics parameters (E_a and *ln(A)*) are evaluated by the peak temperature (T_p) in a series of heating rates (β), Eq. (S6). In Kissinger's method, E_a was considered as a constant during the whole decomposition process.

$$ln\left(\frac{\beta}{{T_p}^2}\right) = ln\left(\frac{AR}{E_a}\right) - \frac{E_a}{RT} \qquad \text{(S6)}$$

According to Friedman's method [5], the E_a is related to conversion α. Since $f(\alpha)$ was considered a constant, the Friedman method is expressed as Eq. (S7). The slope and intersection of the linear regression curve are the values of E_a and *ln (A)*.

$$ln\left(\frac{d\alpha}{dT}\beta\right) = ln(A) + ln\big(f(\alpha)\big) - \frac{E_a}{RT} \qquad \text{(S7)}$$

According to the combined kinetic method [6, 7], Eq. (S8) has been used to describe the ideal model of solid-state processes, autocatalytic equation (Eq. (S3)) was combined with Eq. (S7), and the parameters (*m*, *n*, *A,* and E_a) were calculated and the reaction model was attained.

$$\ln\left[\beta \frac{d\alpha/dT}{\alpha^m(1-\alpha)^n}\right] = \ln(\text{A}) - \frac{E_a}{RT} \qquad \text{(S8)}$$

where $\frac{d\alpha}{dt}$ is the reaction conversion rate; α is the reaction conversion; $k(T)$ is the constant rate; β is the heating rate, K min^{-1}; E_a is the activation energy; T is the temperature, K; $f(\alpha)$ is the reaction model; R the molar gas constant, 8.314 J mol^{-1} K^{-1}; and A is a pre-exponential factor.

2. Thermal properties of involved samples

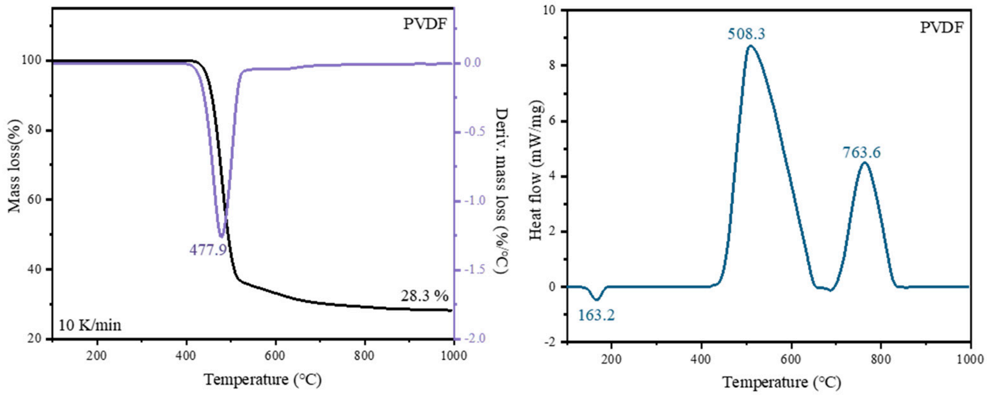

Figure S1. TGA/DTG and DSC of PVDF at 10 K/min.

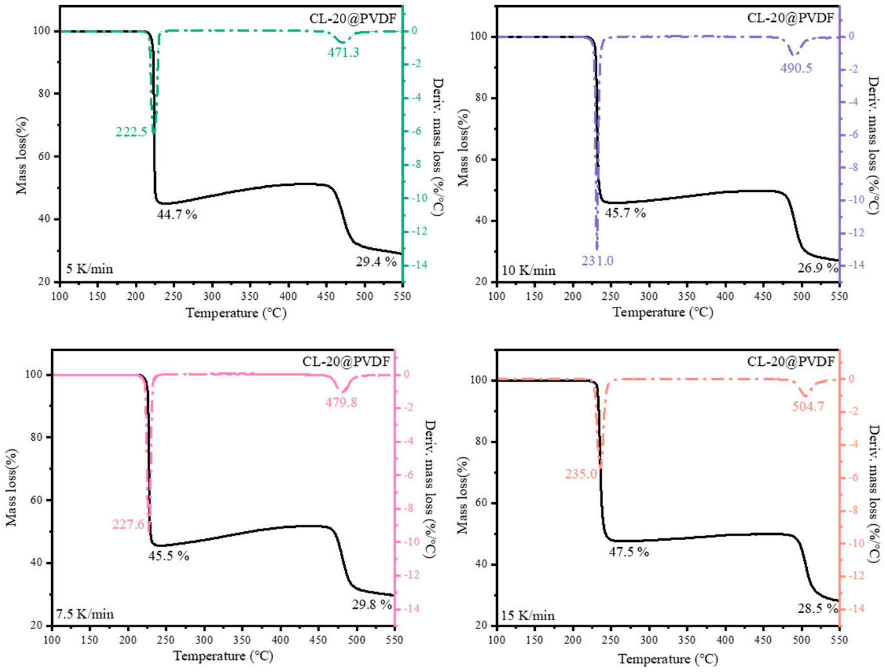

Figure S2. TGA/DTG of CL-20@PVDF microspheres at 5, 10, 7.5, 15 K/min.

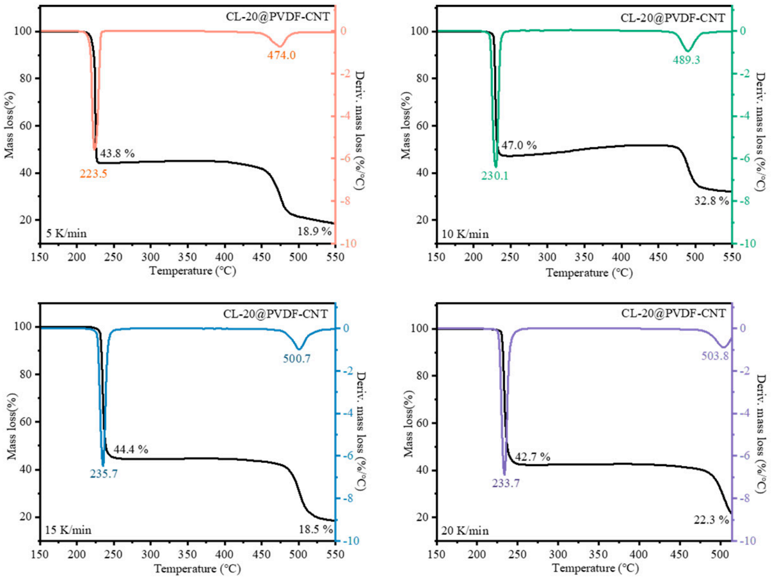

Figure S3. TGA/DTG of CL-20@PVDF-CNT microspheres at 5, 10, 15, 20 K/min.

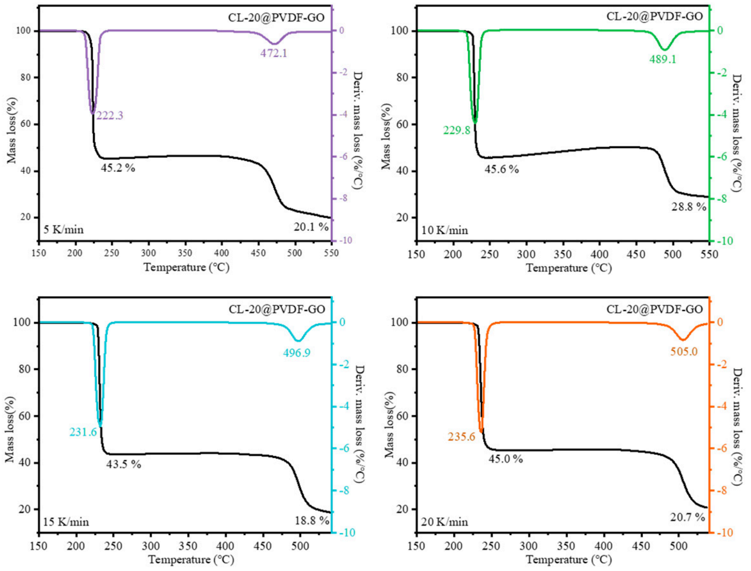

Figure S4. TGA/DTG of CL-20@PVDF-GO microspheres at 5, 10, 15, 20 K/min.

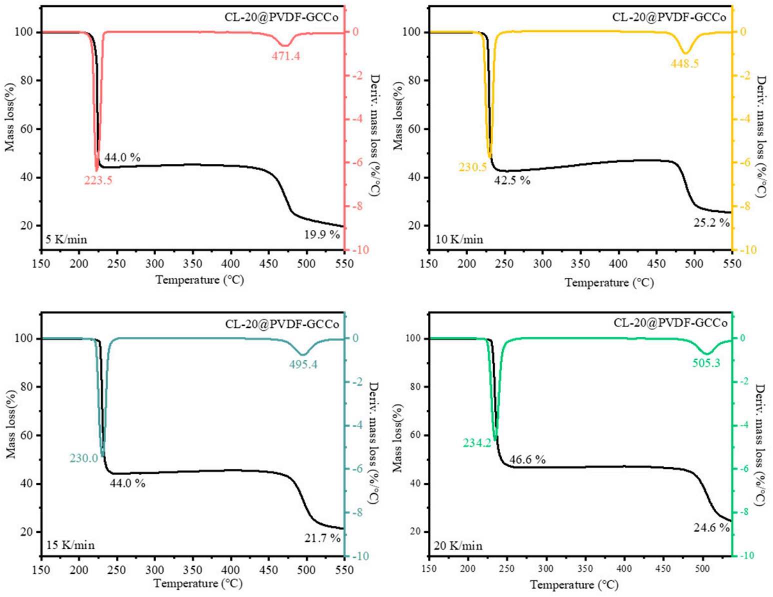

Figure S5. TGA/DTG of CL-20@PVDF-GCCo microspheres at 5, 10, 15, 20 K/min.

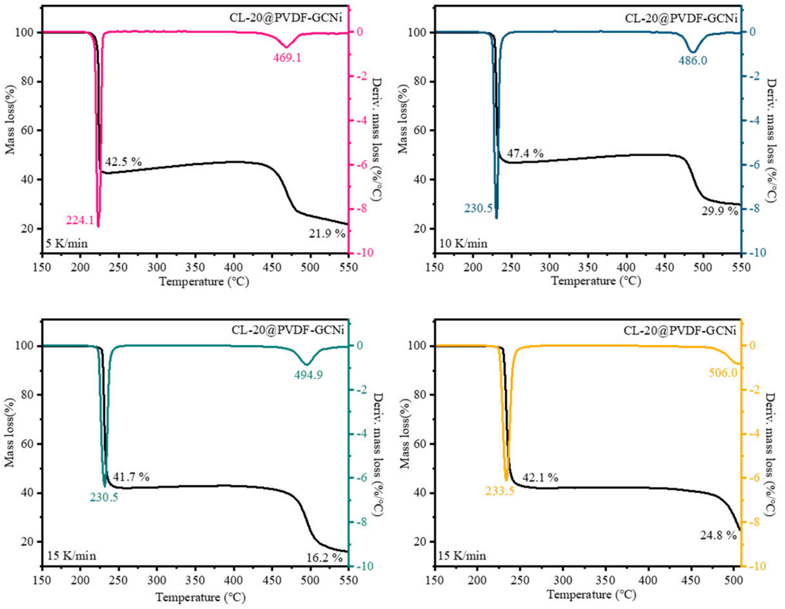

Figure S6. TGA/DTG of CL-20@PVDF-GCNi microspheres at 5, 10, 15, 20 K/min.

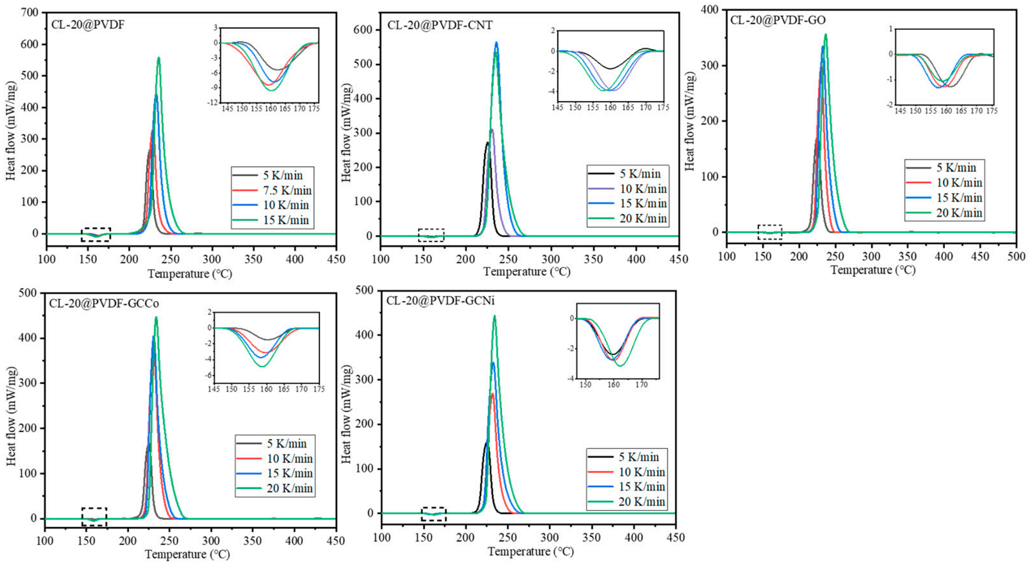

Figure S7. DSC of involved CL-20@PVDF microspheres at 5, 10, 15, 20 K/min.

3. Burn Rate of CL-20@PVDF@B Microspheres

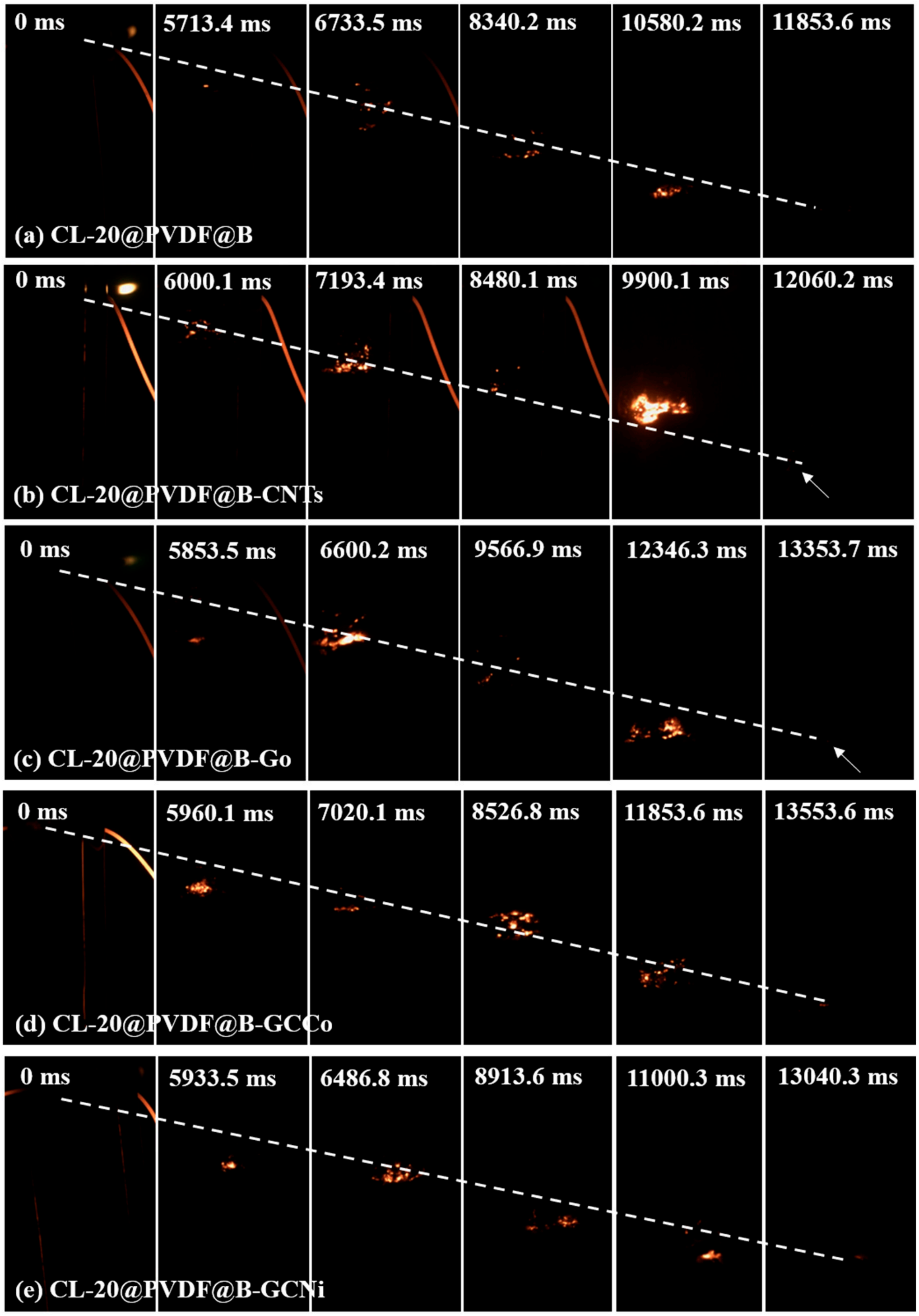

Figure S8. Flame propagation images of the combustion processes of involved CL-20@PVDF@B: (**a**) CL-20@PVDF@B; (**b**) CL-20@PVDF@B-CNTs; (**c**) CL-20@PVDF@B-GO; (**d**) CL-20@PVDF@B-GCCo; (**e**) CL-20@PVDF@B-GCNi.

References

[1] Vyazovkin, S. (2000). Computational aspects of kinetic analysis. Part C. The ICTAC Kinetics Project-The light at the end of the tunnel. Thermochimica Acta, 355(1-2), 155-163-163. https://doi.org/10.1016/S0040-6031(00)00445-7

[2] Jaques, N. G., William de Lima Souza, J., Popp, M., Kolbe, J., Lia Fook, M. V., & Ramos Wellen, R. M. (2020). Kinetic investigation of eggshell powders as biobased epoxy catalyzer. Composites Part B, 183. https://doi.org/10.1016/j.compositesb.2019.107651
[3] Starink, M.. (2003). The determination of activation energy from linear heating rate experiments: a comparison of the accuracy of isoconversion methods. Thermochimica Acta, 404(1), 163–176. https://doi.org/10.1016/S0040-6031(03)00144-8
[4] Blaine, R. L., & Kissinger, H. E. (2012). Homer Kissinger and the Kissinger equation. Thermochimica Acta, 540, 1–6. https://doi.org/10.1016/j.tca.2012.04.008
[5] Friedman, H. L. (1967). Kinetics and Gaseous Products of Thermal Decomposition of Polymers. Journal of Macromolecular Science: Part A-Chemistry, 1(1), 57–79. https://doi.org/10.1080/10601326708053917
[6] Yan, Q.-L., Zeman, S., Svoboda, R., Elbeih, A., & Málek, J. (2013). The effect of crystal structure on the thermal reactivity of CL-20 and its C4-bonded explosives: Part II. Models for overlapped reactions and thermal stability. Journal of Thermal Analysis and Calorimetry: An International Forum for Thermal Studies, 112(2), 837–849. https://doi.org/10.1007/s10973-012-2629-3
[7] Chen, S., He, W., Luo, C.-J., An, T., Chen, J., Yang, Y., Liu, P.-J., & Yan, Q.-L. (2019). Thermal behavior of graphene oxide and its stabilization effects on transition metal complexes of triaminoguanidine. Journal of Hazardous Materials, 368, 404. https://doi.org/10.1016/j.jhazmat.2019.01.073
